# Exploring the role of the *WNT5A* rs566926 polymorphism and its interactions in non-syndromic orofacial cleft: a multicenter study in Brazil

**DOI:** 10.1590/1678-7757-2023-0353

**Published:** 2024-02-12

**Authors:** LARA Lorraynne dos Santos, Ricardo D COLETTA, Renato Assis MACHADO, Lilianny Querino Rocha de OLIVEIRA, Hercílio MARTELLI, Silvia Regina de Almeida REIS, Rafaela SCARIOT, Luiz Evaristo Ricci VOLPATO

**Affiliations:** 1 Universidade de Cuiabá Programa de Pós-Graduação em Ciências Odontológicas Integradas Faculdade de Odontologia de Cuiabá Cuiabá MT Brasil Universidade de Cuiabá, Programa de Pós-Graduação em Ciências Odontológicas Integradas, Faculdade de Odontologia de Cuiabá, Cuiabá, MT, Brasil.; 2 Universidade Estadual de Campinas Programa de Pós-Graduação em Biologia Buco-Dental Faculdade de Odontologia de Piracicaba Piracicaba SP Brasil Universidade Estadual de Campinas, Departamento de Diagnóstico Oral e Programa de Pós-Graduação em Biologia Buco-Dental, Faculdade de Odontologia de Piracicaba, Piracicaba, SP, Brasil.; 3 Universidade Estadual de Campinas Faculdade de Odontologia de Piracicaba Programa de Pós-Graduação em Biologia Buco-Dental Piracicaba SP Brasil Universidade Estadual de Campinas, Faculdade de Odontologia de Piracicaba, Programa de Pós-Graduação em Biologia Buco-Dental, Piracicaba, SP, Brasil.; 4 Universidade Estadual de Montes Claros Departamento de Odontologia Clínica de Estomatologia Montes Claros MG Brasil Universidade Estadual de Montes Claros, Departamento de Odontologia, Clínica de Estomatologia, Montes Claros, MG, Brasil, e; Universidade Professor Edson Antônio Velano Faculdade de Odontologia Centro de Reabilitação de Anomalias Craniofaciais Alfenas MG Brasil Universidade Professor Edson Antônio Velano, Faculdade de Odontologia, Centro de Reabilitação de Anomalias Craniofaciais, Alfenas, MG, Brasil.; 5 Escola Bahiana de Medicina e Saúde Pública Departamento de Ciências Básicas Salvador Bahia Brasil Escola Bahiana de Medicina e Saúde Pública, Departamento de Ciências Básicas, Salvador, Bahia, Brasil.; 6 Universidade Federal do Paraná setor de Ciências da Saúde Departamento de Estomatologia, Disciplina de Cirurgia Bucomaxilofacial Curitiba PR Brasil Universidade Federal do Paraná, setor de Ciências da Saúde, Departamento de Estomatologia, Disciplina de Cirurgia Bucomaxilofacial, Curitiba, PR, Brasil.

**Keywords:** Cleft lip, Cleft palate, Polymorphism, genetic, Wnt5A

## Abstract

Associations between the *WNT5A* rs566926 variant and non-syndromic orofacial cleft (NSOC) have been reported in different populations.

**Objective:**

This study aimed to investigate the role of the rs566926 single nucleotide polymorphism (SNP) in *WNT5A* and its interactions with SNPs in *BMP4, FGFR1, GREM1, MMP2*, and *WNT3* in the occurrence of NSOC in a Brazilian population.

**Methodology:**

A case-control genetic association study was carried out involving participants from four regions of Brazil, totaling 801 patients with non-syndromic cleft lip with or without cleft palate (NSCL±P), 273 patients with cleft palate only (NSCPO), and 881 health volunteers without any congenital condition (control). Applying TaqMan allelic discrimination assays, we evaluated *WNT5A* rs566926 in an ancestry-structured multiple logistic regression analysis, considering sex and genomic ancestry as covariates. Interactions between rs566926 and variants in genes involved in the *WNT5A* signaling pathway (*BMP4, FGFR1, GREM1, MMP2*, and *WNT3*) were also explored.

**Results:**

*WNT5A* rs566926 was significantly associated with an increased risk of NSCL±P, particularly due to a strong association with non-syndromic cleft lip only (NSCLO), in which the C allele increased the risk by 32% (OR: 1.32, 95% CI: 1.04–1.67, p=0.01). According to the proportions of European and African genomic ancestry, the association of rs566926 reached significant levels only in patients with European ancestry. Multiple interactions were detected between *WNT5A* rs566926 and *BMP4* rs2071047, *GREM1* rs16969681 and rs16969862, and *FGFR1* rs7829058.

**Conclusion:**

The WNT5A rs566926 polymorphism was associated with NSCL±P, particularly in individuals with NSCLO and high European ancestry. Epistatic interactions involving *WNT5A* rs566926 and variants in *BMP4, GREM1*, and *FGFR1* may contribute to the risk of NSCL±P in the Brazilian population.

## Introduction

Non-syndromic orofacial cleft (NSOC) is the most common congenital craniofacial malformation and results from incomplete fusion of embryonic facial processes.^1^ The incidence of NSOC varies, with higher rates found in Asian and Native American populations (1:500), followed by European populations (1:1,000), and lower frequencies in populations of African descent (1:2,500).^2^ Due to the highly diverse nature of the Brazilian population, the prevalence of NSOC ranges from 1:650 to 1:2,700 live births, varying across the different states and regions. Notably, the Southern region holds the highest prevalence, whereas the Northeast holds the lowest.^3-5^ The etiology of NSOC involves a complex interplay between genetic factors and environmental exposures.^6^ Several genes and *loci* have been identified as key players in signaling pathways essential for proper lip and palate development during embryogenesis.^7^

The wingless gene family (*WNT*) plays a critical role in cell communication during embryogenesis, regulating processes such as cell growth, motility, and differentiation. In post-embryonic stages, *WNT* genes contribute to tissue homeostasis.^8^ These genes are active in various tissues during craniofacial development and facilitate cell proliferation and polarity.^9^ Disruptions in the regulation of these genes have been associated with developmental defects, including orofacial clefts.^10-13^ Mutations or deletions in member 5A of the WNT family (*WNT5A*) have been associated with an increased risk of NSOC in both animal studies^14^ and population-based investigations.^10,13,15^ *WNT5A* is involved in the regulation of developmental pathways during embryogenesis, such as mesenchymal cell migration in palatogenesis,^15^ and also contributes to postnatal tissue and bone homeostasis.^16^ Due to its broad involvement in several pathways,^17^ *WNT5A* is considered a promising candidate for gene–gene interactions that influence the occurrence of orofacial clefts.^15^ Among the various single nucleotide polymorphisms (SNPs) identified in *WNT5A*, the variant rs566926 has garnered significant attention. It is located in a transcription factor binding site with intronic enhancer function involved in the regulation of embryonic development and cell fate determination^11^ and has been associated with NSOC in European populations, individuals of European descent, and Hispanics.^10,13^

Given the potential role of polymorphic variants in genes associated with craniofacial development in the etiology of NSOC and the possibility of ethnic differences being responsible for the divergences among studies, this study aimed to investigate the association between *WNT5A* rs566926 and NSOC in the Brazilian population. We also investigated SNP–SNP interactions with genes involved in NSOC susceptibility and the *WNT5A* signaling pathway, including *BMP4* (rs11623717, rs17563, rs2071047, and rs2761887), *FGFR1* (rs7829058), *GREM1* (rs16969681, rs16969816, rs16969862, and rs1258763), *MMP2* (rs243836), and *WNT3* (rs11653738).^10,12,18^ The main hypothesis of the study was that the rs566926 polymorphism in the *WNT5A* gene, alone or associated with polymorphisms in its signaling pathway genes, increases the occurrence of NSOC in a Brazilian population.

## Methodology

### Ethical aspects

This multicenter study was conducted in accordance with ethical guidelines and received approval from the research ethics committee of the primary center (approval number: 08452819.0.0000.5418), as well as from the affiliated centers involved in the study. Consent was obtained from the participants and/or their parents or guardians.

### Sample size estimation

The Quanto software (version 1.2.4, https://pphs.usc.edu/biostatistics-software/#quanto) was used to calculate the sample power, ensuring an adequate sample size. Applying specific parameters—case-control design, gene-only analysis, additive inheritance model, genetic effect of 1.28, and minor allele frequency (MAF) of 0.235, which were selected based on the meta-analysis exploring rs566926 in NSOC,^13^ two-sided type I error, and a Brazilian population risk (prevalence) of 0.001459—the sample required for a power of 80% was 677 individuals per group.

### Population

The study included 801 patients with non-syndromic cleft lip with or without cleft palate (NSCL±P), 273 patients with cleft palate only (NSCPO), and 881 individuals in the control group. All participants were enrolled in the Brazilian Oral Cleft Group (BOCG), which is a collaborative group with reference centers for the treatment of patients with orofacial clefts in different geographical regions of Brazil.^25^ The control group consisted of individuals of both sexes who had no family history of orofacial clefts or any congenital abnormalities.

### Selection of genetic polymorphisms

The selection of rs566926 (*WNT5A*) was based on its association with NSOC reported in previous studies.^10,13^ Other SNPs were included due to their potential interactions with *WNT5A*, as determined by the STRING database (http:/string-db.org), and their involvement in signaling pathways related to orofacial cleft development. The SNPs included were *BMP4* rs11623717, rs17563, rs2071047, and rs2761887;^25^ *FGFR1* rs7829058*;*^27^ *GREM1* rs16969681, rs16969816, rs16969862, and rs1258763*;*^23^ *MMP2* rs243836;^26^and *WNT3* rs11653738.^27^

### Genotyping and assessment of genomic ancestry

Genomic DNA was extracted from desquamated oral mucosal cells collected by two methods: rinsing with a 3% sucrose solution or scraping the oral mucosa with a swab. PCR-based genotyping was carried out using the TaqMan^®^ system from Applied Biosystems on the StepOnePlus platform. The genotyping process used TaqMan 5’-exonuclease allelic discrimination assays obtained from the Assay-on-Demand service by Applied Biosystems.

To assess the ancestral background of each individual, 40 biallelic short insertion-deletion polymorphisms (INDELs) previously validated as informative markers of ancestry in the Brazilian population^28^ were included in the analysis. Structure software, version 2.3.4, was employed to determine the genomic ancestry of each participant. The analysis implemented the K = 3 model, considering the tri-hybrid origin of the Brazilian population and following the established model.^29^ This approach allowed the categorization of individuals based on their predominant ancestral components.

### Statistical analysis

The chi-square test was employed to assess the Hardy-Weinberg equilibrium (HWE) of the control group and the differences in sex distribution. The Mann-Whitney test was used to compare the proportions of genomic ancestry between the groups with statistical significance defined as p≤0.05.

Multiple logistic regression analyses were performed using the SNPassoc package in the Rstudio program (JJ Allaire, Boston, Massachusetts, USA). These analyses encompassed unrestricted, dominant, and recessive genetic models, with due consideration of sex and ancestry proportions as potential confounders. Statistical significance after Bonferroni correction for multiple corrections was defined as p≤0.01.

To investigate epistatic interactions, the multifactorial dimensionality reduction test based on the mbmdr package was applied, followed by 1,000 permutations to eliminate false-positive interactions with statistical significance defined as p≤0.05.

## Results

This study involved a total of 1,955 patient samples, with 232 individuals diagnosed with non-syndromic cleft lip only (NSCLO), 568 with non-syndromic cleft lip and palate (NSCLP), 274 with non-syndromic cleft palate only (NSCPO), and 881 controls. The researchers examined individual variations in genetic ancestry proportions and, although some variations were found, the differences were not statistically significant. In all groups, the prevalence of European ancestry was higher compared to African and Amerindian ancestry. Regarding sex, the prevalence of males was significantly higher in NSCL±P (n=451, 56.3%, p<0.0005) and NSCLP (n=326, 57.4%, p<0.0005) compared to the control group (n=421, 47.8%) (Table 1). The genotyping call rate ranged from 97% to 100%. All the genotype frequencies of the control group were in agreement with the HWE, with p-values >0.05, indicating no significant deviations from the expected genetic distribution (Table 2).

**Table 1:** Clinical characteristics of participants

	Control	NSCL±P	NSCLO	NSCLP	NSCPO
	**(n=881)**	**(n=801)**	**(n=233)**	**(n=568)**	**(n=273)**
**Sex**					
Male	421 (47.8%)	451 (56.3%)*	125 (53.6%)	326 (57.4%)*	105 (38.5%)
Female	460 (52.2%)	350 (43.7%)	108 (46.4%)	242 (42.6%)	168 (61.5%)
**Ancestry**					
European	83.4%	82.7%	82.4%	82.7%	80.9%
African	14.8%	15.4%	15.9%	15.3%	16.6%
Amerindian	1.8%	1.9%	1.7%	2.0%	2.5%

* Statistically different from the control group at p<0.0005

NSCL±P: non-syndromic cleft lip with or without cleft palate; NSCLO: non-syndromic cleft lip only; NSCLP: non-syndromic cleft lip and palate; NSCPO: non-syndromic cleft palate only

**Table 2:** Characteristics of single nucleotide polymorphisms (SNPs), frequency of minor alleles, Hardy-Weinberg equilibrium and genotyping rate

Gene	SNP	Position	Allele	MAF	Call Rate	HWE (p-value)
*WNT5A* (chr 3)	rs566926	55486750	A/c	0.322	97.0%	0.72
*FGFR1* (chr 8)	rs7829058	38474577	G/c	0.186	100.0%	0.07
*BMP4* (chr 14)	rs11623717	53947414	A/g	0.359	98.5%	0.38
	rs17563	53950804	A/g	0.325	99.2%	0.10
	rs2071047	53951693	G/a	0.410	98.5%	0.78
	rs2761887	53958334	C/a	0.588	99.2%	0.51
*GREM1* (chr 15)	rs16969681	32700910	C/t	0.197	99.9%	0.49
	rs16969816	32701444	G/a	0.324	99.8%	0.12
	rs16969862	32701800	A/c	0.222	99.6%	0.37
	rs1258763	32758222	C/t	0.459	99.5%	0.39
*MMP2* (chr 16)	rs243836	55500324	G/a	0.464	98.8%	0.74
*WNT3* (chr 17)	rs11653738	46809587	T/c	0.264	100.0%	0.49

Minor allele in bold MAF: Minor allele frequency

The distribution of alleles and genotypes of rs566926 are shown in Tables 3, 4, and 5. The C allele was associated with NSCLO (OR_Allele_:1.32; 95% CI:1.04–1.67; p=0.01), leading to a potential association with NSCL±P (OR_Allele_:1.18; 95% CI: 1.00–1.39; p=0.03) (Table 3). Other potential associations at a nominal p-value were observed in the CC genotype with NSCL±P (OR: 1.47; 95% CI: 0.98–2.22; p=0.05) and NSCLO (OR: 1.87; 95% CI: 1.07–3.28; p=0.03). In the dominant model, rs566923 suggests an association with NSCLO (OR_Dom_: 1.34; 95% CI: 1.00–1.81; p=0.05) (Table 3). The distribution of alleles and genotypes was further investigated considering the genomic ancestry of the individuals. In individuals with high European genomic ancestry, the C allele was associated with NSCL±P (OR_Allele_: 1.25 (95% CI: 1.04–1.51; p=0.01). Potential associations were observed with NSCLO (OR_Allele_:1.32; 95% CI: 1.00–1.74; p=0.04) and NSCLP (OR_Allele_: 1.22; 95% CI: 0.99–1.50; p=0.05) (Table 4). No associations were identified among individuals with high African genomic ancestry (Table 5).

**Table 3:** Association between polymorphism in rs566926 - *WNT5A* and the occurrence of non-syndromic cleft lip with or without cleft palate (NSCL±P), non-syndromic cleft lip only (NSCLO), non-syndromic cleft lip and palate (NSCLP), and non-syndromic cleft palate only (NSCPO). P-values were adjusted for covariates by logistic regression analysis and corrected by Bonferroni correction

	Control %	NSCL±P %	OR (95% CI)/ p-value	NSCLO %	OR (95% CI)/ p-value	NSCLP %	OR (95% CI)/ p-value	NSCPO %	OR (95% CI)/ p- value
Allele									
A	77.1	73.9	Reference	71.7	Reference	74.9	Reference	76.2	Reference
C	22.9	26.1	1.18 (1.00-1.39)/0.03	28.3	1.32 (1.04-1.67)/0.01	25.1	1.12 (0.94-1.34)/0.18	23.8	1.05 (0.83-1.31)/0.68
Genotype									
AA	59.6	55.4	Reference	52.5	Reference	56.7	Reference	59.6	Reference
AC	34.9	37.0	1.13 (0.92-1.40)/0.24	38.6	1.26 (0.92-1.73)/0.15	36.3	1.08 (0.86-1.37)/0.50	33.2	0.94 (0.70-1.27)/0.68
CC	5.5	7.6	1.47 (0.98-2.22)/0.05	9.0	1.87 (1.07-3.28)/0.03	7.0	1.32 (0.84-2.09)/0.21	7.2	1.36 (0.77-2.40)/0.29
Dominant (AA/AC+CC)	59.6/40.4	55.4/44.6	1.18 (0.97-1.44)/0.10	52.5/47.5	1.34 (1.00-1.81)/0.05	56.7/43.3	1.12 (0.89-1.39)/0.33	59.6/40.4	0.99 (0.75-1.32)/0.96
Recessive (AA+AC/CC)	94.5/5.5	92.4/7.6	1.40 (0.94-2.09)/0.09	91.0/9.0	1.70 (0.99-2.94)/0.06	93.0/7.0	1.28 (0.82-2.01)/0.27	92.8/7.2	1.39 (0.80-2.43)/0.25

**Table 4:** Association between polymorphism in rs566926 - *WNT5A* and occurrence of non-syndromic cleft lip with or without cleft palate (NSCL±P), non-syndromic cleft lip only (NSCLO), non-syndromic cleft lip and palate (NSCLP), and non-syndromic cleft palate only (NSCPO) in individuals stratified by high European genomic ancestry. P-values were adjusted for covariates by logistic regression analysis and corrected by Bonferroni correction

	Control %	NSCL±P %	OR (95% CI)/ p-value	NSCLO %	OR (95% CI)/ p-value	NSCLP %	OR (95% CI)/ p-value	NSCPO %	OR (95% CI)/ p-value
Allele									
A	76.8	72.6	Reference	71.5	Reference	73.0	Reference	73.5	Reference
C	23.2	27.4	1.25 (1.04-1.51)/0.01	28.5	1.32 (1.00-1.74)/0.04	27.0	1.22 (0.99-1.50)/0.05	26.5	1.19 (0.91-1.55)/0.18
Genotype									
AA	59.6	5.1	Reference	51.5	Reference	53.7	Reference	56.1	Reference
AC	34.4	39.0	1.25 (0.98-1.60)/0.07	39.9	1.34 (0.93-1.93)/0.13	38.7	1.22 (0.93-1.60)/0.14	34.9	1.06 (0.74-1.50)/0.76
CC	5.9	7.9	1.45 (0.91-2.30)/0.11	8.6	1.64 (0.85-3.18)/0.15	7.6	1.36 (0.82–2.26)/0.24	9.0	1.56 (0.84–2.89)/0.19
Dominant (AA/AC+CC)	59.6/40.4	53.1/46.9	1.28 (1.02-1.62)/0.03	51.5/48.5	1.38 (0.98-1.95)/0.06	53.7/46.3	1.24 (0.96-1.60)/0.09	56.1/43.9	1.13 (0.81-1.57)/0.47
Recessive (AA+AC/CC)	94.1/5.9	92.1/7.9	1.32 (0.84-2.08)/0.22	91.4/8.6	1.46 (0.77-2.77)/0.25	92.4/7.6	1.25 (0.76-2.07)/0.37	91.0/9.0	1.52 (0.84-2.78)/0.18

**Table 5:** Association between rs566926 - W*NT5A* polymorphism and the occurrence of non-syndromic cleft lip with or without cleft palate (NSCL±P), non-syndromic cleft lip only (NSCLO), non-syndromic cleft lip and palate (NSCLP), and non-syndromic cleft palate only (NSCPO) in individuals stratified by high African genomic ancestry. P-values were adjusted for covariates by logistic regression analysis and corrected by Bonferroni correction

	Control %	NSCL±P %	OR (95% CI)/ p-value	NSCLO %	OR (95% CI)/ p-value	NSCLP %	OR (95% CI)/ p-value	NSCPO %	OR (95% CI)/ p-value
Allele									
A	77.7	77.8	Reference	72.5	Reference	80.0	Reference	82.9	Reference
C	22.3	22.2	0.99 (0.71-1.38)/0.98	27.5	1.32 (0.83-2.09)/0.23	20.0	0.86 (0.59-1.25)/0.45	17.1	0.72 (0.44-1.16)/0.17
Genotype									
AA	59.5	62.1	Reference	55.0	Reference	65.2	Reference	68.4	Reference
AC	36.4	31.3	0.84 (0.56-1.28)/0.41	35.0	1.09 (0.59-2.02)/0.82	29.7	0.76 (0.48-1.21)/0.23	28.9	0.68 (0.38-1.22)/0.18
CC	4.1	6.6	1.49 (0.61-3.63)/0.35	10.0	2.57 (0.85-7.81)/0.09	5.1	1.11 (0.40-3.10)/0.82	2.6	0.61 (0.13-2.95)/0.51
Dominant (AA/AC+CC)	59.5/40.5	62.1/37.9	0.91 (0.61-1.36)/0.65	55.0/45.0	1.25 (0.70-2.23)/0.45	65.2/34.8	0.80 (0.51-1.24)/0.31	68.4/31.6	0.67 (0.38-1.18)/0.16
Recessive (AA+AC/CC)	95.9/4.1	93.4/6.6	1.58 (0.66-3.80)/0.30	90.0/10.0	2.49 (0.84-7.37)/0.11	94.9/5.1	1.22 (0.44-3.36)/0.70	97.4/2.6	0.69 (0.14-3.31)/0.63

Lara LS, Coletta RD, Machado RA, Oliveira LQ, Martelli Júnior H, Reis SR, Scariot R, Volpato LE

A pairwise analysis was conducted to assess the effects of interactions between *WNT5A* rs566926 and the SNPs of the other genes studied (*BMP4, FGFR1, GREM1, MMP2*, and *WNT3*) on the risk of NSOC (Table 6). These analyses revealed associations of SNPs that interacted with *WNT5A* after correcting the p-values using a permutation test. The largest interaction was found with the *BMP4* rs2071047 SNP, which was significantly associated with NSCL±P (p=0.03) and NSCLO (p=0.02); the interaction between *WNT5A* (rs566926) and *BMP4* (rs2761887) was associated with NSCLO (p=0.05). Interactions involving SNPs in *GREM1* were exclusively associated with the occurrence of NSCL±P (p=0.05 for rs566926–rs16969681 and p=0.04 for rs566926–rs16969862). The interaction between *WNT5A* (rs566926) and *FGFR1 (*rs7829058) was (p=0.04) only for the occurrence of NSCLP.

**Table 6:** Gene–Gene interactions between *WNT5A* and *BMP4, GREM1, MMP2*, and *WNT3* assessed by the model-based multifactor dimensionality reduction (mbmdr) test

	SNP1	SNP2	NH^**a**^	betaH^**b**^	NL^**c**^	betaL^**d**^	p-value^**e**^	Perm. P-value^**f**^
NSCL±P	rs566926 (*WNT5A*)	rs2071047 (*BMP4*)	1	13.357	1	-0.2479	2.13e-03	**0.03**
	rs566926 (*WNT5A*)	rs16969681 (*GREM1*)	1	0.6185	0	-	7.82e-03	**0.05**
	rs566926 (*WNT5A*)	rs16969862 (*GREM1*)	1	0.6231	0	-	8.14e-03	**0.04**
	rs566926 (*WNT5A*)	rs11623717 (*BMP4*)	1	0.8021	0	-	2.58e-02	0.21
	rs566926 (*WNT5A*)	rs1258763 (*GREM1*)	1	0.6355	1	-0.3667	4.57e-02	0.32
	rs566926 (*WNT5A*)	rs16969816 (*GREM1*)	1	0.5046	0	-	5.38e-02	0.33
	rs566926 (*WNT5A*)	rs243836 (*MMP2*)	0	-	1	-0.2047	6.77e-02	0.34
NSCLP	rs566926 (*WNT5A*)	rs7829058 (*FGFR1*)	2	0.3338	2	-0.5739	0.006	**0.04**
	rs566926 (*WNT5A*)	rs2071047 (*BMP4*)	1	11.687	1	-0.2821	0.01	0.12
	rs566926 (*WNT5A*)	rs16969681 (*GREM1*)	1	0.5504	0	-	0.03	0.17
NSCLO	rs566926 (*WNT5A*)	rs2071047 (*BMP4*)	1	16.480	0	-	0.001	**0.02**
	rs566926 (*WNT5A*)	rs2761887 (*BMP4*)	2	0.9896	1	-0.4462	0.002	**0.05**
	rs566926 (*WNT5A*)	rs243836 (*MMP2*)	0	-	1	-0.4675	0.009	0.09
	rs566926 (*WNT5A*)	rs16969681 (*GREM1*)	1	0.7645	0	-	0.01	0.10
	rs566926 (*WNT5A*)	rs16969862 (*GREM1*)	1	0.7203	1	- 0.5429	0.02	0.16
NSCPO	rs566926 (*WNT5A*)	rs2761887 (*BMP4*)	1	0.3755	2	-0.4430	0.003	**0.04**
	rs566926 (*WNT5A*)	rs2071047 (*BMP4*)	2	0.4912	0	-	0.01	0.17

^a^Number of significant high-risk genotypes in the interaction ^b^Regresion coeficient in step2 for high-risk exposition ^c^Number of significant low-risk genotypes in the interaction ^d^Regresion coeficient in step2 for low-risk exposition ^e^P-value for the interaction model adjusted for covariates ^f^Permutation p-value for the interaction model

## Discussion

The *WNT5A* rs566926 variant was significantly associated with NSCLO in the Brazilian population studied, confirming the study hypothesis. In individuals with high European ancestry, the rs566926 SNP was associated with the occurrence of NSCL±P. In contrast, no significant association was identified in individuals with high African genomic ancestry. Interactions between *WNT5A* rs566926 and SNPs in *BMP4, GREM1*, and *FGFR1* reached significant levels in the NSOC population.

Our results are consistent with data described in previous studies, which identified an association between the *WNT5A* rs566926 SNP and NSOC in individuals of European-American and European descent.^10,13^ The association between rs566926 and NSOC suggests a potential role for *WNT5A* in craniofacial development. The *WNT* signaling pathway is involved in several developmental processes, including facial morphogenesis.^30,31^ *WNT5A* has been implicated in craniofacial development and has been shown to play a role in regulating cell migration, cell polarity, and tissue morphogenesis.^15,32^ The specific mechanisms by which rs566926 influences NSOC susceptibility are not yet fully understood and require further investigation. However, one possibility is interference with the migration of mesenchymal cells during palatogenesis.^15^

Epistatic interactions between genes contribute to the complexity of multifactorial diseases, such as NSOC. Several epistatic interactions involving the *WNT5A* rs566926 SNP were identified in this study. Strong interactions were observed between rs566926 and *BMP4* (rs2071047 and rs2761887) SNPs, which are important for craniofacial development.^33^ *BMP4* is a member of the bone morphogenetic protein family and has been shown to be involved in facial development, including palate fusion.^34,35^ The interaction between *WNT5A* and *BMP4* suggests a synergistic effect on NSOC susceptibility, highlighting the intricate interplay between these genes in craniofacial development.^15,35^ Another notable interaction identified in this study was between *WNT5A* and *GREM1. GREM1* is a regulator of bone morphogenetic protein (BMP) signaling that has been implicated in craniofacial development.^20^ Although the precise mechanisms underlying the interaction between *WNT5A* and *GREM1* remain unclear, it is likely that they modulate common signaling pathways involved in craniofacial morphogenesis. This study also identified an interaction between the *WNT5A* rs566926 and *FGFR1* rs7829058, which revealed a protective association for NSCLP. *FGFR1* encodes fibroblast growth factor receptor 1, which is involved in various cellular processes, including craniofacial development.^36^ The protective association observed in this interaction suggests a potential compensatory effect between *WNT5A* and *FGFR1* in NSCLP susceptibility. On the other hand, no significant associations were found between the *WNT5A* rs566926 SNP and the *MMP2* or *WNT3* SNPs in the occurrence of NSOC. This is consistent with data from previous studies that did not identify a correlation between *MMP2* and NSOC in the Brazilian population.^27,37^ The relationship between *WNT3* and NSOC remains uncertain and requires further investigation.

The most popular classification divides NSOC into three main subtypes, NSCLO, NSCLP, and NSCPO.^25,38^ Although not universally accepted, due to similarities in epidemiological characteristics and embryological timing, many studies combine NSCLO and NSCLP into a single group: NSCL±P.^38,39^ Therefore, the analyses were carried out with these subgroups. The association between the polymorphism in rs566926 – *WNT5A* was found only in the NSCLO group, influencing a potential association with a nominal p-value in the NSCL±P group. Although *WNT* expression is observed in the upper lip and primary and secondary palates,^10,30^ these structures differ in their embryonic origins.^39^ This may justify the association between rs566926 – *WNT5A* only with the NSCLO group. However, *WNT5A* is expressed in the frontonasal prominence and maxillary processes, which fuse to form the primary palate.^10^ Therefore, it is possible that we did not find differences in the NSCLP and NSCPO groups due to the limited sample size in these subgroups.

The ethnic diversity of a population must be considered in NSOC studies^40^. This is because prevalence of different types of clefts can vary in the population when comparing an immigrant group with the country’s local ethnic group, as the immigrant group tends to have a similar prevalence of NSOC to the ethnic groups in their countries of origin.^25,39^ Brazilians have a wide range of genomic ancestry, with varying proportions of European, African, and Amerindian ancestries.^29^ This diversity significantly influences genetic susceptibility of NSOC.^40^ Therefore, to avoid population stratification bias, we took into account the variation in the genetic ancestry of each individual. The association found only in the group with high European ancestry may be due to the higher proportion of individuals with this ancestry in the sample, but also because populations with predominantly European ancestry have a higher incidence of NSOC compared to those with predominantly African ancestry.^2^

In addition to the aforementioned reduced strength of subgroup samples, it is important to note that this study employed a case-control design, which holds certain limitations. One potential limitation is the heterogeneity of the case and control groups, as different genes may carry alleles with protective or risk associations, depending on the ethnic ancestry of the population.^23^ To address this concern, the researchers sought to create homogeneous case and control groups in terms of ancestry. The predominant ancestry in both groups was European, followed by African and Amerindian, which reflects the known ancestry distribution in the Brazilian population.^29^ However, it is important to acknowledge that there may still be underlying genetic heterogeneity within these broad ancestral categories. Future studies should incorporate a larger sample of individuals with NSCLP and NSCPO and explore gene–environment interactions. Additionally, these studies should strive to include individuals from all regions of Brazil, focusing particularly on those with greater representation of African and Amerindian ancestry to further explore genetic associations with NSOC.

## Conclusion

In summary, this study found that the *WNT5A* rs566926 variant is associated with NSCL±P in the Brazilian population, with the strongest association found in individuals with NSCLO. The association was particularly significant in individuals with high European ancestry. The researchers also found that interactions between *WNT5A* rs566926 and SNPs in the *BMP4, GREM1*, and *FGFR1* genes were significantly associated with NSOC, indicating their combined influence on the occurrence of the condition. These findings provide valuable insights into the genetic factors contributing to NSOC and emphasize the importance of considering genetic interactions and genetic diversity in the population to understand the complex nature of this condition.
